# Using Microbiological Sampling to Evaluate the Efficacy of Nasofibroscope Disinfection: The Tristel Trio Wipes System in Ear–Nose–Throat (ENT) Endoscopy

**DOI:** 10.3390/ijerph16224583

**Published:** 2019-11-19

**Authors:** Savina Ditommaso, Monica Giacomuzzi, Raffaella Cipriani, Teresa Zaccaria, Rossana Cavallo, Valeria Boggio, Roberto Albera, Carla M. Zotti

**Affiliations:** 1 Department of Public Health and Pediatrics, University of Turin, 10126 Turin, Italy; monica.giacomuzzi@unito.it (M.G.); carla.zotti@unito.it (C.M.Z.); 2 Laboratory of Microbiology and Virology, Città della Salute e della Scienza Hospital, 10126 Turin, Italy; raffaella.cipriani23@gmail.com (R.C.); tzaccaria@cittadellasalute.to.it (T.Z.); 3 Department of Public Health and Pediatrics, University of Turin, Laboratory of Microbiology and Virology, Città della Salute e della Scienza Hospital, 10126 Turin, Italy; rossana.cavallo@unito.it; 4 Department of Surgical Sciences, Otolaryngology Unit, University of Turin, 10126 Turin, Italy; valeboggio87@gmail.com (V.B.); roberto.albera@unito.it (R.A.)

**Keywords:** flexible fiberoptic laryngoscope, disinfection, sampling, wipes

## Abstract

Disinfection and sterilization are needed for guaranteeing that medical and surgical instruments do not spread contagious microorganisms to patients. The aim of this study was to evaluate the efficacy of a simple manual technique of high-level disinfection (HLD) of flexible fiberoptic nasofibroscopes (FFNs) with wipes impregnated with a chlorine dioxide solution (Tristel Trio Wipes System—TTW) against a conventional automated washer machine (Soluscope ENT, Cimrex 12—AW). FFNs used in 62 patients undergoing endoscopy at an ENT clinic were sampled according to an aseptic procedure. For each nasoendoscopy, microbiological samples were taken at two times: (1) after a patient’s nasoendoscopy and (2) immediately after high-level disinfection. Ten microliters of each prepared sample were inoculated onto specific culture media for the detection of nasopharyngeal flora microorganisms. The microbiological results obtained from 62 post-disinfection samples revealed bacterial growth on two FFNs disinfected with AW, and five FFNs disinfected with TTW, but this difference is not statistically significant. None of the isolates were pathogenic bacteria. Our results are different than the results obtained by two previously published studies on the TTW system. In both studies, sampling was carried out by swabbing the tip and the handle surface of FFNs. This sampling method was the least effective method means of detecting bacteria on a surface. It can be concluded that the two disinfection systems allow providers to obtain a reduction of the saprophytic and pathogenic microbial load.

## 1. Introduction

Surgeries and other invasive medical procedures entail physical contact between a surgical or medical instrument and a patient’s sterile tissue or mucous membranes. A great risk of all such procedures is the presence of pathogens that can cause infection. Disinfection and sterilization are needed for guaranteeing that medical and surgical instruments do not spread contagious microorganisms to patients [1].

The classification system suggested by Earle H. Spaulding separates medical devices into three categories based on the risk of infection in relation to their utilization [2]. This classification establishes which level of disinfection would be proper for various different kinds of devices that are reused. The classification is based on the possible risk of infection posed to a patient and largely recognized and applied by the U.S. Food and Drug Administration (FDA), the Centers for Disease Control and Prevention (CDC), the European health authorities such as the Italian Ministry of Health, microbiologists, epidemiologists, and professional medical organizations.

According to Spaulding’s scheme, the flexible fiberoptic nasofibroscopes (FFNs) are considered a semi-critical device because they are “a device that comes in contact with undamaged membranes or non-intact skin”. Therefore, these devices should be given at least high-level disinfection (HLD), specified as the destruction of all vegetative microorganisms, mycobacteria, small or nonlipid viruses, medium or lipid viruses, fungal spores and some, but not all, bacterial spores” [3].

FFNs are normally contaminated by microorganisms residing in the upper aerodigestive tract. The use of inappropriate systems of cleaning, disinfection or sterilization may increase the risk of disease transmission from patient to patient. Case reports involving possible cross-infection of laryngoscopes with *Listeria monocytogenes* [4] and *Pseudomonas aeruginosa* [5,6] have been described. However, while few reports of nosocomial infections linked to inadequately disinfected FFNs have been published, these events may go unrecognized or may not be recorded [3].

There are difficulties related with high-level disinfection. Traditionally, FFNs were subjected to manual disinfection procedures but these methods are frequently time-wasting, potentially damaging to the instrument in question and dangerous to the healthcare personnel. 

Currently, there is no uniformity in the recommendations; many guidelines require a centralized area for HLD and automated washer-machines to minimize the chemical and infectious risks for health personnel and guarantee the standardization of procedures [7].

The chemicals used in some automated mechanical washers may increase the risk of damaging the optical fibers of FFNs.

Several chemicals have good disinfection properties, including chlorine dioxide, hypochlorous acid/superoxidized water and peracetic acid. Peracetic acid is an irritant to exposed skin and the respiratory system. Glutaraldehyde is no longer in use as it carries high risks of inducing sensitivity. Orthophthalaldehyde (OPA) has begun to take the place of glutaraldehyde in many hospitals because it does not irritate the eyes and the nasal mucosa, it does not need activation or monitoring during disinfection and it only takes 12 min to effectively sterilize.

Flexible endoscopes cannot resist the high temperatures and pressure variations in autoclave cycles and thus, are normally decontaminated by cleaning, followed by high-level disinfection (HLD) with a chemical sterilant.

The CDC [1] recommends HLD with glutaraldehyde-based formulas (2%), ortho-phthalaldehyde (0.55%), stabilized hydrogen peroxide (6%), peracetic acid or wet pasteurization. The FDA recommendations for heat-labile devices employ “low temperature” reprocessing techniques, including ozone (O_3_) sterilization, hydrogen peroxide (H_2_O_2_) sterilization, ethylene oxide (EO) sterilization (including device aeration) and liquid chemical sterilant/high level disinfectant chemical systems [8]. 

Other methods of disinfection, such as immersion in enzymatic soap solution, chlorine dioxide wipes, and isopropyl alcohol washes are acceptable alternatives but have not been employed widely for different reasons, such as the lack of FDA registration or the fact that some hospitals prefer to introduce central decontamination models with a standardized decontamination program (where the responsibility for decontamination is outsourced) or the use of automated washer-machines (AWs), common in every endoscopy clinic.

Automated washer machines provide several advantages [9] such as:

✓ patient and personnel safety
✓ a high level of standardization in reprocessing
✓ documentation of several important endoscope reprocessing parameters (printed automatically)
✓ audible and visual alarms activated when a safety fault is detected
✓ a lower work-load compared to full manual reprocessing.

However, some disadvantages [9] related to the use of AWs must also be considered, such as:

✓ the specialized AWs require a separate reprocessing room
✓ if AWs are not kept properly, they may themselves become an infection hazard by contamination of endoscopes during reprocessing. Systematic maintenance and validation of is compulsory
✓ potentially high costs
✓ the endoscopy procedure may have to be cancelled if AWs break down
✓ the washing process may decrease the clarity of the optical image within a short period of time.

The aim of this study was to evaluate the efficacy of a simple manual technique of HLD of FFNs with wipes impregnated with a chlorine dioxide solution (Tristel Trio Wipes System) against a conventional AW, Soluscope ENT Cimrex 12, in accordance with standard practices in the otolaryngology clinic of the hospital. 

## 2. Materials and Methods

The FFNs used in 62 patients undergoing endoscopy at an ENT clinic at the City of Health and Science Hospital were attributed to one of two groups by alternating group placement (i.e., group AW, group TTW, group AW). The FFNs are cleaned and disinfected in the same area of ENT clinic. 

The Ethics Committee of the City of Health and Science Hospital ruled that non-formal ethics approval was required because the research was not considered a human-subject research.

### 2.1. Automated Mechanical Washer (AW)

Soluscope ENT Cimrex 12 (Soluscope SAS, Aubagne, France) are designed for decontaminating 3 flexible endoscopes simultaneously during each 14 min cycle. FFNs are processed in single-use chemical products.

The disinfection with the AW system is carried out as follows:

1. acidic pre-disinfectant cleaner, compatible with peracetic acid; then
2. five minutes of high-level disinfection with a 5% peracetic acid-based disinfectant with anticorrosive additive; then
3. rinsing with filtered water (0.2 µm) to ensures that bacteria free water is supplied to the FFNs.

At the end of the washing cycle, endoscopes were placed in a separate room for drying and stored for no more than 72 h.

### 2.2. Tristel Trio Wipes (TTW)

The chlorine dioxide manual wipe system “Tristel Trio Wipes” (Tristel plc, Cambridgeshire, U.K.) is a three-part decontamination system for non-lumened medical devices. It is comprised of three wipes that are used to perform the steps of the decontamination procedure in a matter of minutes. The disinfection of the nasendoscope with the TTW system is carried out as follows [10]:

1. pre-cleaning with the Tristel Pre-Clean Wipe,
2. high-level disinfection with the sporicidal wipe for a contact time of 30 s,
3. neutralization of any chemical residues with the rinse wipe.

Hospital ENT staff were specifically trained to operate according to standard instructions given by the manufacturer. This model of disinfection has not yet been adopted by the Food and Drug Administration (FDA), but it has been approved and is in common use in Oceania and Europe. Currently, TTW is widely known and adopted in England as advised by ENT UK [11].

Following this experimental decontamination, all FFNs were decontaminated according to the hospital’s standard operating procedure with Soluscope ENT prior to clinical use because Soluscope ENT is the standard method of the hospital.

### 2.3. Sampling Procedure

We developed a surface sampling method (by TNT wipes) to detect low levels of bacteria on a FFNs as discussed in a recent publication [12].

Two laboratory personnel who were familiar with the instruments and proficient in aseptic technique performed the sampling protocol. One person held the FFNs, while the other person extracted samples accordingly.

For each nasoendoscopy, microbiological samples were taken at two times: (1) after a patient’s nasoendoscopy and (2) immediately after high-level disinfection. 

**Figure 1:**
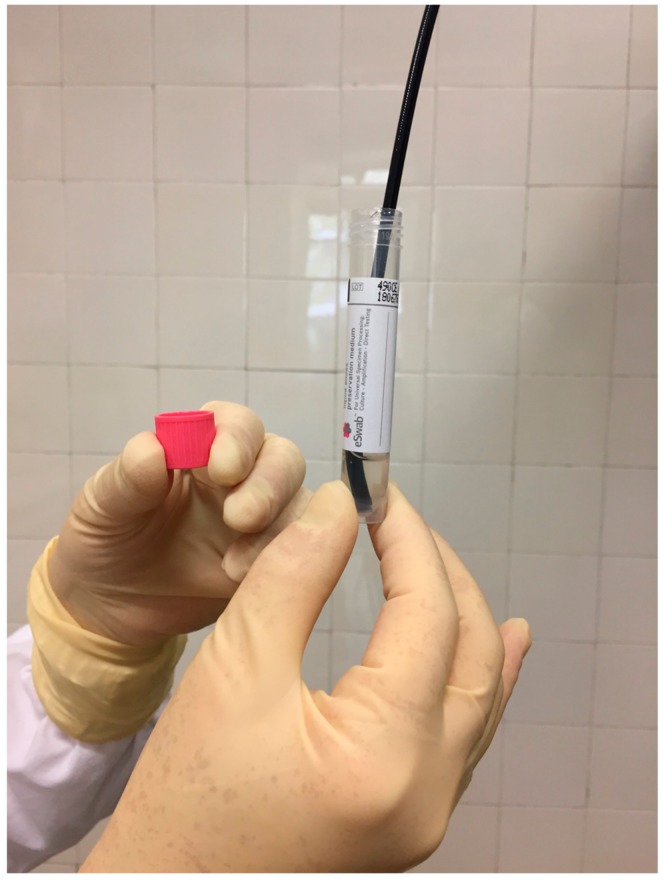
Sampling after the patient’s nasoendoscopy.

The first sample was carried out by shaking the optic tip of the insertion shaft of the FFL in 2 mL of in a sterile collection tube with liquid Amies preservation medium (Copan, Brescia, Italy) (Figure 1).

The pre-disinfection samples were not obtained with the TNT wipes, thereby avoiding the bioburden from the devices that had to be disinfected.

**Figure 2:**
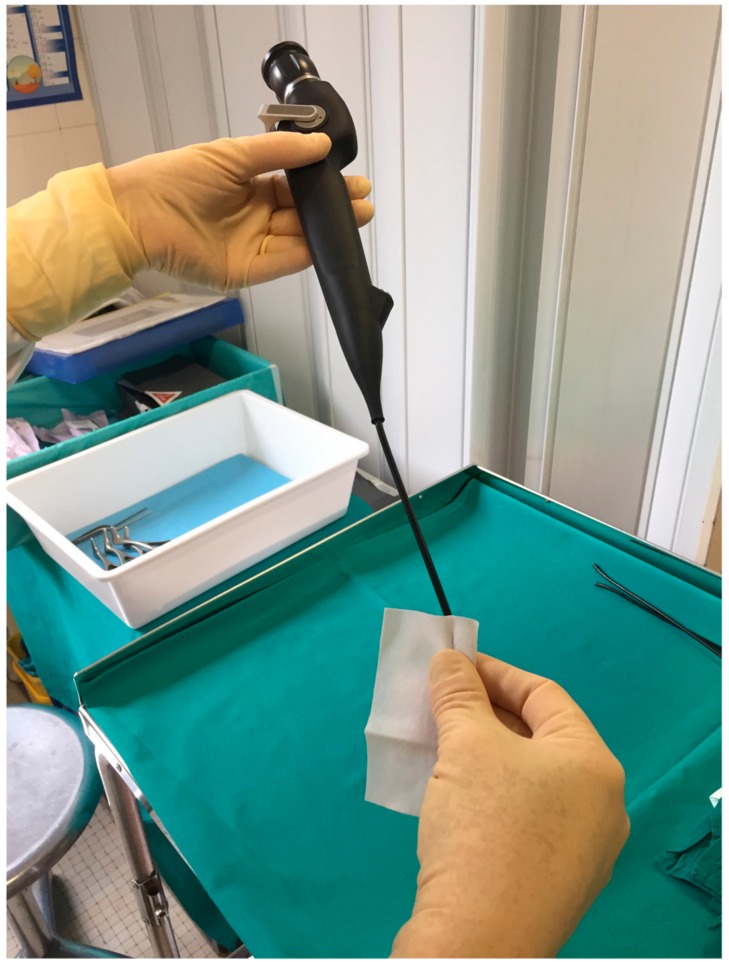
Sampling after high-level disinfection.

The second sample was collected using the wipe procedure [12]. We sampled the whole insertion shaft of the FFL with a sterile TNT wipe pre-moistened with 0.5 mL of sterile water by wiping the surface from top to bottom of the insertion tube, folding the exposed side of the wipe to the interior, and wiping once again (Figure 2).

Next, each wipe was eluted in 20 mL of sterile Page’s buffer.

Following each sampling run, pre- and post-disinfection samples were delivered to the microbiology laboratory for preparation and standard culture.

### 2.4. Laboratory Analysis

#### 2.4.1. Sample Preparation

1. The pre-disinfection samples (tube with 2 mL of Page’s rinsing solution) were vortexed for 30 s.
2. The post-disinfection samples were vortexed for 30 sec in order to extract the microbial load from the wipe, then centrifuged (3000× *g* for 30 min) and finally suspended in 2 mL of Page’s buffer.

#### 2.4.2. Culture and Identification

Ten microliters of each prepared sample were inoculated onto specific culture media for the detection of the main microorganisms of the nasopharyngeal flora.

Aerobic cultures were incubated at 37 °C in a standard atmosphere and examined after 24–48 h; anaerobic cultures were incubated in jars filled with mixed gas at 37 °C for 7 days with examination every 48 h. 

The numbers of bacterial colonies on each media were added to obtain total colony-forming units (CFU).

The isolated aerobic and anaerobic bacteria were identified by means of the mass spectrometry (MALDI-TOF) technique [13,14].

### 2.5. Statistical Analysis

The Mann-Whitney U test was adopted to evaluate the between-group differences (AW vs. TTW) in the cell counts in pre-disinfection samples. A Chi-squared test was run to determine whether there is a significant difference between bacterial growth status and cleaning method.

## 3. Results

Altogether, we analyzed 124 samples, of which 62 samples were collected from FFNs before disinfection, 31 after disinfection with AW and 31 after disinfection with TTW.

All of the samples obtained in the pre-disinfection phase yielded aerobic bacteria by culture.

Major components of Gram-positive normal nasal flora (coagulase-negative staphylococci, *S. aureus*, *Corynebacterium* spp., and streptococci of the viridans group) were recovered in 100% of the samples. Gram-negative bacteria, belonging to the families *Enterobacteriaceae*, *Neisseriaceae* and of the genus *Pseudomonas* and *Haemophilus* were recovered in 42% of samples analyzed. *Candida albicans* was isolated in two samples. Table 1 summarizes the microbial results.

There were no differences between the two sample groups in terms of the isolation rates of Gram-positive, Gram-negative bacteria or fungi.

To assess the quantity of bacterial growth in the pre-disinfection samples, quantitative culture results of the two groups were estimated and compared. The differences in isolation counts were not significant (*p* = 0.9681) (Table 2).

After disinfection, seven samples returned positive cultures. Thus, the bacterial recovery rate from FFNs was 11.3%. Two samples (6.5%) from FFNs disinfected with AW disinfection grew cultures of *S. viridans*, *S. epidermidis* and *Rothia mucillaginosa*. Five samples (16.1%) from FFNs disinfected with TTW grew culture of *S. viridans*, *S. epidermidis, S. hominis* and *Micrococcus luteus*. No significant difference was observed between the two study groups in terms of disinfection efficacy (*chi-square* test = 1.4494; *p* = 0.4248; IC 95% = 0.5241–11.92). Table 3 summarizes the pre- and post-disinfection bacteria isolations.

In both groups, few colonies of microorganisms belonging to the normal oro-pharyngeal flora were isolated; no oral and nasal pathogens, such as *H. influenza*, *S. pneumoniae*, and *P. aeruginosa*, were recovered after the high-level disinfection procedure.

## 4. Discussion

The risk of transmission of pathogenic or opportunistic microorganisms from water or preceding patients through endoscopic instruments is documented in the literature [4,5,15,16,17,18,19,20]. Opportune reprocessing of FFNs and endoscopic accessories are an extremely important part of quality assurance and patient safety.

Disinfection and sterilization are extremely important for guaranteeing that medical and surgical instruments do not convey infectious pathogens to patients. Because sterilization of medical devices is not always necessary, healthcare policies must identify whether cleaning, disinfection, or sterilization is indicated.

This study was undertaken to evaluate the microbicidal efficacy of Tristel Trio Wipes vs. automated washer machine (Soluscope ENT Cimrex 12) in disinfection of FFNs. 

The microbiological results obtained from 62 post-disinfection samples revealed bacterial growth on two FFNs disinfected with AW, and five FFNs disinfected with TTW, but this difference is not statistically significant. None of the isolates were pathogenic bacteria. In six samples out of seven, colony counts ranged from 1 to 5 CFU; only in one sample obtained from TTW group after disinfection were the colony counts 600 CFU of *Micrococcus luteus* and *S. epidermidis*.

The limitation of this study is the small sample size; we analyzed only 62 samples to avoid spending too many resources, e.g., time and financial costs. The data from this research should be used to design a larger confirmatory study.

Our results are different than the results obtained by two previously published studies on the TTW system. In one study [21], 31 cleaning episodes with the TTW system were monitored, and no samples developed any organism growth. The study of Hitchcock [7] compared the microbiological efficacy of TTW vs two other high-level disinfection systems, and no positive results were returned from 72 FFNs treated with TTW.

In both studies, no paired samples (after patient’s nasoendoscopy and immediately after high-level disinfection) were taken, and baseline microbiological contamination was not known. Furthermore, sampling was carried out by swabbing the tip and the handle surface of FFNs. This sampling method was the least effective method of detecting bacteria on a surface with very variable recovery rates [22].

In our study, we adopted TNT wipes, a more efficient device for sampling surfaces that could improve the accuracy and reproducibility of environmental surveillance as discussed in a previous study [12].

Sampling with the TNT wipes allowed us to recover more microorganisms and thus, to find (contrary to the other studies) five positive post-disinfection samples, though these had low bacterial charges.

CDC guidelines [23] advise microbiological culture surveillance as an “unresolved issue” for the following reasons: (1) it is confounded by frequent isolation of nonpathogenic organisms from skin or environmental contamination; (2) cultures are not validated by correlating viable counts on an endoscope with the development of an infection; (3) false-positive and false-negative rates and limits of detection have not been established; (4) negative culture does not guarantee effective processing; (5) sensitivity of routine cultures may not be reliable for detecting organisms associated with outbreaks; (6) the need to quarantine endoscopes (until culture results have been obtained) does not allow for rapid reuse and could lead to delays in patient care; (7) it is resource-intensive and requires additional expenses for testing and time for personnel to collect and process samples; and (8) it is impractical for facilities that do not have access to microbiology laboratories. 

Despite these arguments, there are guidelines for sampling and interpretation of detectable bioburden on channeled instruments, such as flexible gastrointestinal endoscopes and bronchoscopes [24,25,26] but there are no shared instructions for instruments without channels. In otorhinolaryngology, to date, no specific references exist.

## 5. Conclusions

Considering that the disinfection of FFNs is a complex and poorly regulated field, it can be concluded that the two disinfection systems compared herein allow providers to obtain a reduction of the saprophytic and pathogenic microbial load. Since the upper airways normally have a saprophytic microbial flora and no invasive procedures are performed that alter the integrity of the mucosa during the endoscopic investigation, the examination with a flexible endoscope does not require sterile conditions. The finding of non-pathogenic saprophyte microorganisms, even after disinfection with TTW, does not compromise the use of the instrument in normal outpatient clinical practice.

However, as advised in guidelines of several international organizations for channeled endoscopes, microbiological culture surveillance can be used to monitor the effectiveness and quality of processing. However, it remains necessary to determine benchmarks for microbial levels. 

## Figures and Tables

**Table 1:** Culture results of 62 pre-disinfection samples.

Bacterium	*No.* of Samples with the Indicated Isolate (%)
*Staphylococcus epidermidis*	45 (73)
*Staphylococcus aureus*	23 (37)
*Coagulase* *-negative staphylococci (CoNS)*	8 (13)
*Streptococcus viridans*	27 (44)
*Streptococcus pneumoniae*	5 (8)
*Corynebacterium spp*	19 (31)
*Corynebacterium striatum*	3 (5)
Other gram-positive bacteria	10 (16)
*Pseudomonas aeruginosa*	7 (11)
*Stenotrophomonas maltophilia*	1 (2)
*Neisserie*	6 (10)
*Haemophilus influenzae*	5 (8)
*Haemophilus parainfluenzae*	8 (13)
*Enterobacteriaceae*	8 (13)
Non-fermenting Gram-negative bacilli	2 (3)
Other Gram-negative bacteria	1 (2)
*Candida albicans*	2 (3)

**Table 2:** Quantitative results obtained by culture of pre-disinfection samples.

Type of Samples	TVCs (Geometric Mean CFU ± ds)
AW	2.54 × 10^2^ ± 5.42 × 10^2^
TTW	2.41 × 10^2^ ± 4.71 × 10^2^

*U*-value = 477.5 The *p*-value is 0.9681 (Mann–Whitney). TVCs: total viable counts; CFU: colony forming unit; AW: Automated Mechanical Washer; TTW: Tristel Trio Wipes.

**Table 3:** Culture results of 7 positive samples.

Code/Groups	Bacterium (Colony Count)
24 AW pre-disinfection	*Corynebacterium* spp., *S. epidermidis* (≅100) *
24 AW post-disinfection	*Kokuria* spp. (2)
42 AW pre-disinfection	*S. viridans*, *S. epidermidis* (≅200) *
42 AW post-disinfection	*Rothia mucillaginosa* (4)*, S. viridans* (1)
1 TTW pre-disinfection	*S. epidermidis*, *S. viridans*, *H. parainflenzae*, *N. subflava* (≅400) *
1 TTW post-disinfection	*S. hominis* (1)
15 TTW pre-disinfection	*S. epidermidis* (100)
15 TTW post-disinfection	*S. epidermidis* (1)
22 TTW pre-disinfection	*S. epidermidis* (6)
22 TTW post-disinfection	*S. epidermidis* (1)
23 TTW pre-disinfection	*S. aureus, S. epidermidis* (≅500) *
23 TTW post-disinfection	*S. epidermidis* (2)
26 TTW pre-disinfection	*S. aureus*, *C. striatum*, *P.aeruginosa*, *S. epidermidis*, *S. viridans* (≅1300) *
26 TTW post-disinfection	*Micrococcus luteus, S. viridans* (≅600) *

* total colony count.
